# Genetic Polymorphisms in the Glutathione S-transferase Gene With the Association of Lung Cancer: A Hospital-Based Case-Control Study in Southwestern Maharashtra

**DOI:** 10.7759/cureus.112436

**Published:** 2026-07-10

**Authors:** Nilam Jagdale, Satish Patil, Pratik Durgawale, Satish V Kakade, Rashmi A Gudur, Aishwarya P Patil

**Affiliations:** 1 Department of Molecular Biology and Genetics, Krishna Institute of Medical Sciences, Krishna Vishwa Vidyapeeth (Deemed to be University), Karad, IND; 2 Department of Microbiology, Krishna Institute of Medical Sciences, Krishna Vishwa Vidyapeeth (Deemed to be University), Karad, IND; 3 Department of Community Medicine, Krishna Institute of Medical Sciences, Krishna Vishwa Vidyapeeth (Deemed to be University), Karad, IND; 4 Department of Oncology, Krishna Institute of Medical Sciences, Krishna Vishwa Vidhyapeeth (Deemed to be University), Karad, IND; 5 Department Molecular Biology and Genetics, Krishna Institute of Medical Sciences, Krishna Vishwa Vidhyapeeth, (Deemed to be University), Karad, IND

**Keywords:** gstm1, gstp1, gstt1, lung cancer, pcr-rflp

## Abstract

Background

Lung cancer is one of the leading causes of cancer-related mortality globally. Genetic polymorphisms in detoxification genes such as glutathione S-transferase genes *GSTM1*, *GSTT1*, and *GSTP1 *(exon 5 and exon 6) may influence individual susceptibility to lung cancer by modulating the capacity to eliminate carcinogens.

Objective

The study aims to evaluate the association of *GSTM1*, *GSTT1*, and *GSTP1 *(exon 5 and exon 6) gene polymorphisms with lung cancer in a hospital-based population in southwestern Maharashtra.

Methods

A total of 460 participants (230 histopathologically confirmed lung cancer patients and 230 age- and sex-matched healthy controls) were enrolled. Genomic DNA was extracted and genotyped for *GSTM1*, *GSTT1 *(deletion polymorphism), and *GSTP1 *exon 5 and exon 6 (*SNPs*) using PCR and *PCR-RFLP*. Statistical analysis included Chi-square test and odds ratios (OR) with 95% confidence intervals (CI).

Results

The *GSTT1 *null genotype was positively associated with increased susceptibility to lung cancer (<0.001). The *GSTM1 *null genotype and *GSTP1 *(exon 5) heterozygous variant were negatively associated with a protective effect. *GSTP1 *(exon 6) polymorphism did not show any significant correlation.

Conclusion

The findings suggest that polymorphisms in *GSTT1*, *GSTM1*, and *GSTP1 *genes contribute to lung cancer susceptibility in the southwestern Maharashtra population.

## Introduction

Lung cancer is one of the most common malignancies in India and continues to be the primary cause of death from cancer globally. Its incidence continues to rise due to increasing tobacco consumption and environmental exposures. Approximately 90% of lung cancer cases are attributed to tobacco use, making it a well-established risk factor for the disease. Tobacco smoke contains more than 50 established carcinogens, including polycyclic aromatic hydrocarbons, aromatic amines, and tobacco-specific nitrosamines. Key culprits include polycyclic aromatic hydrocarbons (PAHs), nitroso compounds, aromatic amines, and the potent tobacco-specific nitrosamine 4-(methylnitrosamino)-1-(3-pyridyl)-1-butanone (NNK)* *[1]. Once inhaled, these carcinogens undergo metabolic activation and detoxification through the body’s xenobiotic metabolism pathways.

Deoxyribonucleic acid adducts can result from activated carcinogens forming covalent bonds with DNA. If left untreated, these adducts could result in somatic mutations that start the carcinogenic process. A class of phase II detoxification enzymes called glutathione S-transferases (GSTs) is essential for neutralizing and removing these toxic intermediates from cells [2].

The GST gene superfamily is responsible for detoxifying aromatic and electrophilic compounds [3, 4]. Detoxification occurs in two main enzymatic phases: Phase I enzymes, such as the cytochrome P450 family, activate or convert procarcinogens into reactive intermediates [5]. Other metabolic enzymes, including CYP1A1, CYP2A6, NAT2, and EPHX1, also contribute significantly to the metabolism of tobacco-derived carcinogens and may influence individual susceptibility to lung cancer [6]. Phase II enzymes, including GSTM1, GSTT1, and GSTP1, conjugate these intermediates with glutathione, facilitating their excretion [7]. The glutathione S-transferase superfamily provides essential protection against toxic compounds [8, 9]. Deletion polymorphisms in these genes, particularly *GSTM1 *and *GSTT1*, have been associated with several cancers, including lung [9,10], bladder [11], colon [12], and skin cancer [13].

The *GSTM1 *gene, located on chromosome 1p13, exists in two main forms: *GSTM1-null *(*GSTM1-0*), a homozygous deletion resulting in complete loss of enzyme activity (nonfunctional type), and *GSTM1-present *(*GSTM1-1*), carrying at least one functional allele (*GSTM1a *or *GSTM1b*) [14].

Homozygous deletion of the *GSTM1 *gene results in loss of enzyme function, impairing the detoxification of reactive carcinogenic metabolites. Consequently, individuals with the *GSTM1-null *genotype, particularly smokers, tend to accumulate higher concentrations of PAH-DNA adducts in lung tissue, which may promote genomic instability and increase susceptibility to lung cancer [15, 16]. Similarly, the *GSTT1 *gene plays an important role in the detoxification of halogenated compounds and reactive oxygen species, and its deletion (null genotype) can also elevate cancer susceptibility.

The most common GST enzyme in the lung, GSTP1, detoxifies various carcinogens, such as benzo(a)pyrene found in tobacco smoke. However, genetic variations in *GSTP1* exons 5 (Ile105Val) and 6 (Ala114Val) cause amino acid changes that alter enzyme function and reduce its ability to clear these toxins [17]. Individuals carrying the variant alleles (105Val or 114Val) may have reduced enzymatic activity, leading to accumulation of DNA-damaging compounds and an elevated risk of lung cancer.

However, susceptibility to lung cancer is not determined solely by carcinogen metabolism. DNA repair pathways also play a critical role in maintaining genomic integrity by removing tobacco-induced DNA damage. Genes involved in base excision repair and nucleotide excision repair, such as *XRCC1*, *OGG1*, and *ERCC2*, influence the persistence of DNA adducts and may modulate individual cancer risk through variations in DNA repair capacity [18].

Although numerous studies have investigated the relationship between GST polymorphisms and lung cancer risk, the reported associations have generally been modest, and findings across different populations have sometimes been inconsistent. These discrepancies may reflect ethnic differences, variation in environmental exposures, limited sample sizes, smoking habits, and gene-environment interactions [19].

Given these findings, it is evident that genetic variations in *GSTM1*, *GSTT1*, and *GSTP1 *genes can modify individual susceptibility to lung cancer, particularly among smokers. Understanding these polymorphisms in specific populations is crucial for identifying genetic risk factors and potential biomarkers. Hence, the present case-control study aims to investigate the association of *GSTT1*, *GSTM1*, and *GSTP1 *(exon 5 and exon 6) polymorphisms with lung cancer risk in the southwestern Maharashtra population, where both genetic variability and tobacco exposure may contribute significantly to disease development.

## Materials and methods

Study subjects

A hospital-based case-control study was conducted involving 230 clinically confirmed lung cancer patients and 230 healthy, disease-free controls frequency-matched for age and sex. Participants were recruited from Krishna Hospital and Research Center, Karad, Maharashtra, India, from January 2022 to December 2024. Participants aged 18-75 years were enrolled in the study. Cases and controls were interviewed regarding socioeconomic status, dietary habits, family history, tobacco and alcohol consumption status, and other confounding risk factors. The Modified Kuppuswamy Socioeconomic Status Scale was used for classification based on monthly family income, education of the head of the family, and occupation of the head of the family. The subjects in the cases and control groups were divided into ("Poor," "Middle Class," and "Rich”). The classification "Poor" is a grouping together of two socioeconomic classes - Lower class and Upper lower class; "middle class" is a grouping together of lower middle class and upper middle class; and "Rich" is the upper class according to the updated Kuppuswamy socioeconomic categories [20]. Data were collected using a structured questionnaire (see Appendices). The study protocol was approved by the Institutional Ethics Committee of Krishna Institute of Medical Sciences, Karad (Approval No. 012/2021-2022). Written informed consent was obtained from all participants prior to enrolment.

The sample size was calculated by the following formula: 

\[ n=\frac{\left[(p_1q_1)+(p_2q_2)\right]\left(Z_{1-\alpha/2}+Z_{1-\beta}\right)^2}{(p_1-p_2)^2} \]

Genomic DNA isolation from blood

Genomic DNA was isolated from samples of peripheral blood from cases and controls by using the Omega Bio-tek E.Z.N.A. Blood DNA Mini Kit (Cat. No. D3392-02) (Omega Bio-tek Inc, Norcross, USA) following the manufacturer’s instructions. The quality and quantity of DNA were determined by agarose gel electrophoresis. The DNA samples were stored at -80°C until further use.

Genotyping assay

The *GSTP1* exon 5 (Ile105Val) gene PCR reaction mixture (20 µl) contained 5 ρM of each primer sense (5′GTA GTT TGC CCA AGG TCA AG-3′) and antisense (5′-AGC CAC CTG AGG GGT AAG-3′), 100 ng of genomic DNA, 1.5 mM MgCl2, 2.5 mM each dNTPs, and 1 U Taq DNA polymerase (GeNei Laboratories, Bengaluru, India ). Amplification was performed with an initial denaturation at 95°C for 5 min, followed by 35 cycles at 95°C for 30 sec, 72°C for 30 sec, 52°C for 30 sec, and a final extension at 72°C for 5 min. After confirmation of the expected 433 bp PCR amplification product by agarose gel electrophoresis, the PCR products were digested with 3 U of the restriction enzyme BsmAI (New England BioLabs, Hitchin, UK) at 37°C overnight. The restriction fragment length polymorphism (RFLP) products were separated by electrophoresis on a 3% agarose gel, stained with ethidium bromide, and visualized under a ultraviolet (UV) transilluminator. Gel images were captured using a gel documentation system (Bio-Rad Laboratories, Hercules, USA ). The expected fragment sizes were 328 bp and 105 bp for the wild-type genotype, 328 bp, 222 bp, 106 bp, and 105 bp for the heterozygous genotype, and 222 bp, 106 bp, and 105 bp for the mutant genotype.

The *GSTP1 *exon 6 (Ala114Val) polymorphism was carried out in a total reaction volume of 20 µL containing 100 ng of genomic DNA, 5 pM of each primer (forward: 5′-GGG AGC AAG CAG AGG AGA AT -3′ and reverse: 5′-CAG GTT GTA GTC AGC GAA GGA G-3′), 1.5 mM MgCl₂, 2.5 mM of each dNTP(deoxynucleoside triphosphate), and 1 U of Taq DNA polymerase (GeNei Laboratories). PCR amplification was initiated with a denaturation step at 95°C for 5 min, followed by 30 cycles consisting of denaturation at 95°C for 30 s, annealing at 57°C for 30 s, and extension at 72°C for 30 s, with a final extension at 72°C for 5 min. After the confirmation of an amplified fragment of the expected size (420 bp) on an agarose gel, the PCR products were digested with 5 U of restriction enzyme AciI (New England BioLabs) at 37°C for overnight incubation. The expected fragment sizes were: wild type 246 bp, 116 bp, 58 bp, heterozygous 362 bp, 246 bp, 116 bp, 58 bp, mutant 362 bp, 58 bp. The results were checked using 3% agarose gel electrophoresis, and the gel image was captured by a gel documentation system (Bio-Rad Laboratories).

Genotyping of the *GSTM1* and *GSTT1 *genes was performed using the polymerase chain reaction (PCR) technique. PCR amplifications for GSTM1 and GSTT1 were carried out separately in a total reaction volume of 20 µL containing 1× PCR buffer, 0.2 mM of each dNTP, 10 pmol of each primer (Xcelris Genomics, Ahmedabad, India), 1 U of Taq DNA polymerase (GeNei), and 100 ng of genomic DNA. The primers used for amplification of *GSTM1* were: forward primer 5′-GAA CTC CCT GAA AAG CTA AAG C-3′ and reverse primer 5′-GTT GGG CTC AAA TAT ACG GTG G-3′, while for *GSTT1 *the primers were: forward primer 5′-TTC CTT ACT GGT CCT CAC ATC TC-3′ and reverse primer 5′-TCA CCG GAT CAT GGC CAG CA-3′.

Polymerase chain reaction amplification of the 215 bp *GSTM1 *fragment was performed with an initial denaturation at 95 °C for 5 min, followed by 35 cycles of denaturation at 95 °C for 30 s, annealing at 54 °C for 30 s, and extension at 72 °C for 30 s, with a final extension at 72 °C for 5 min. For *GSTT1*, amplification of the 480 bp fragment was carried out under similar conditions, except that the annealing temperature was 60 °C.

All PCR reactions were performed using a Mastercycler Nexus Gradient thermal cycler (Eppendorf, Hamburg, Germany). Amplified DNA fragments were analyzed by electrophoresis on agarose gels prepared in tris acetate-EDTA (TAE) ​buffer, followed by staining with ethidium bromide (10 mg/mL) and visualization using a UV transilluminator. Gel images were documented using a gel documentation system (Bio-Rad Laboratories). The homozygous null (non-functional) genotypes of *GSTT1 *and *GSTM1 *were identified by the absence of the corresponding amplification products, whereas the presence of the gene was confirmed by detection of the specific PCR fragments.

Statistical analysis

The association between polymorphisms in the *GSTP1*, *GSTM1*, and *GSTT1 *genes and lung cancer risk was initially evaluated using the chi-square test to compare genotype distributions between cases and controls. Odds ratios (ORs) with 95% confidence intervals (CIs) were calculated using GraphPad InStat software (GraphPad, San Diego, USA). To account for potential confounding factors, adjusted odds ratios were estimated using binary logistic regression analysis. Variables entered into the regression model included smoking status and intensity, socioeconomic status, alcohol consumption, family history of cancer, and occupational exposure, where applicable. Although cases and controls were frequency-matched for age and sex, residual confounding from unmeasured variables cannot be completely excluded.

## Results

Demographic and lifestyle characteristics

Comparison of demographic characteristics revealed no significant differences in age and gender between cases and controls (p = 0.956 and p = 0.99, respectively), demonstrating successful matching of participants. However, a significant association was observed with economic status (χ² = 147.3, p < 0.05), with a higher proportion of cases belonging to the poor socioeconomic group (51.3%) compared to controls (15.2%) (Table 1).

**Table 1:** Distribution of selected demographic characteristics of lung cancer cases and healthy controls from rural areas of southwestern Maharashtra, India Socioeconomic status was classified using the Modified Kuppuswamy Scale (family income, education, and occupation of the head of the family) [20]. Categories were regrouped as: "Poor" (lower and upper lower classes), "Middle Class" (lower middle and upper middle classes), and "Rich" (upper class). Significance is at p<0.05.

Variables		Cases (n=230)	Controls (n=230)	Chi-squared value	p-value
Age	Up to 40 years	14 (6.1%)	12 (5.2%)	0.319	0956
	41-50 years	39 (17%)	40 (17.4%)		
	51-60 years	71 (30.9%)	75 (32.6%)		
	61 years or older	106 (46.1%)	103 (44.8%)		
Gender	Male	142 (61.7%)	130 (58.7%)	0.54	0.99
	Female	88 (38.3%)	100 (40.3%)		
Socioeconomic status	Poor (Lower class - Upper lower class)	118 (51.3%)	35 (15.2%)	147.3	<0.05
	Middle class (Lower middle class - Upper middle class)	110 (47.8%)	89 (38.7%)		
	Rich (Upper class)	2 (0.9%)	106 (46.1%)		
Education	Illiterate	79 (34.3%)	80 (34.8%)	1.407	0.704
	High school	115 (50%)	106 (46.1%)		
	Higher secondary	15 (6.5%)	16 (7%)		
Diet	Vegetarian	39 (17%)	73 (31.7%)	13.64	<0.05
	Mixed	191 (83%)	157 (68.3%)		
Family history of cancer	Yes	20 (8.7%)	1 (0.4%)	18.03	<0.05
	No	210 (91.3%)	229 (99.6%)		
Smoking habit	Yes	48 (20.9%)	5 (2.2%)	39.43	<0.05
	No	182 (79.1%)	225 (97.8%)		
Tobacco chewing habit	Yes	141 (61.3%)	15 (6.5%)	153.9	<0.05
	No	89 (38.7%)	215 (93.5%)		
Drinking habit	Yes	45 (19.6%)	3 (1.3%)	41.03	<0.05
	No	185 (80.4%)	227 (98.7%)		

Dietary habits also showed a significant association (χ² = 13.64, p < 0.05), with mixed-diet individuals predominant among cases (83%) compared to controls (68.3%) (Table 1).

A positive family history of cancer was observed in 8.7% of cases and only 0.4% of controls (χ² = 18.03, p < 0.05), indicating a potential hereditary predisposition.

Among behavioural risk factors, significant associations were observed with smoking habit (χ² = 39.43, p < 0.05), tobacco chewing habit (χ² = 153.9, p < 0.05), and alcohol consumption (χ² = 41.03, p < 0.05). The prevalence of smoking was 20.9% among cases compared to 2.2% among controls, while tobacco chewing was reported in 61.3% of cases versus 6.5% of controls. Similarly, 19.6% of cases reported alcohol consumption compared to 1.3% of controls (Table 1).

Distribution of GST genotypes and association with lung cancer risk 

GSTP1 *(exon 5) polymorphism*

The wild-type genotype was predominant in both cases (87.8%) and controls (73.5%). The frequency of the heterozygous genotype was significantly lower among cases (11.7%) compared to controls (26.5%). The heterozygous genotype demonstrated a significant negative association with lung cancer risk (OR = 0.37, 95% CI = 0.22-0.61, p < 0.001). The variant genotype was rare (0.4%) and did not show a statistically significant association, likely due to its low frequency. After adjustment for potential confounders, the heterozygous genotype remained significantly associated with a reduced risk of lung cancer, suggesting a possible protective effect.

GSTP1 *(exon 6) polymorphism*

For *GSTP1 *(exon 5), the wild-type genotype was found in 70.4% of cases and 60.4% of controls. The heterozygous genotype was observed in 26.1% of cases and 34.3% of controls, showing no significant association (OR = 0.65, 95% CI = 0.43-0.97, p = 0.25). The variant genotype (3.5% in cases and 5.2% in controls) also showed no significant difference (OR = 0.57, 95% CI = 0.22-1.44,p = 0.25). After adjustment for confounders, no significant association was observed. 

GSTM1 *polymorphism*

A significant difference was observed in the distribution of *GSTM1 *genotypes between cases and controls. The null (absent) genotype was found in 13.9% of cases compared to 30% of controls. The *GSTM1 *null genotype showed a protective association with lung cancer (OR = 0.24, 95% CI = 0.15-0.39, p < 0.001). After adjustment, this association remained significant (adjusted OR = 0.293, 95% CI = 0.188-0.455, p < 0.05).

GSTT1 *polymorphism*

A strong and statistically significant association was found between the *GSTT1 *null genotype and lung cancer susceptibility. The null genotype was observed in 79.1% of cases and 23.5% of controls (χ² = 38.63, p < 0.001). The *GSTT1 *null genotype was associated with a markedly increased risk of lung cancer (OR = 12.36, 95% CI = 7.9-19.2, p < 0.001), which was not significant after adjustment (adjusted OR = 1.095, 95% CI = 0.629-1.903, p =0.749).

The distribution of *GSTP1*, *GSTM1*, and *GSTT1 *(exon 5 and exon 6) polymorphisms among cases and controls and their corresponding odds ratios are shown in Table 2.

**Table 2:** Genotype distribution of GSTM1, GSTT1, and GSTP1 gene variants in lung cancer cases and healthy controls OR, odds ratio; CI, confidence interval; 1, reference.

Gene	Genotype	Cases (n=230)	Controls (n=230)	Chi- square	p-value	OR (95% Cl)	p-value	Adjusted OR (95% Cl)	p-value
*GSTP1* (exon 5)	Wild type	202 (87.8%)	169 (73.5%)	5.15	0.076	1		1	
	Heterozygous	27 (11.7%)	61 (26.5%)			0.37 (0.22-0.61)	<0.001	1.586 (0.903-2.787)	0.109
	Variant type	1 (0.4%)	0 (0.0%)			2.5 (0.1-62.1)	1	16.76 (0)	0.99
*GSTP1 *(exon 6)	Wild type	162 (70.4%)	139 (60.4%)	17.07	<0.001	1		1	
	Heterozygous	60 (26.1%)	79 (34.3%)			0.65 (0.43-0.97)	0.25	0	0.99
	Variant type	8 (3.5%)	12 (5.2%)			0.57 (0.22-1.44)	0.25	0.203 (0.13-0.318)	<0.05
GSTM1	Present	198 (86.1%)	157 (68.3%)	38.63	<0.001			1	
	Absent	32 (13.9%)	69 (30%)			0.24 (0.15-0.39)	<0.001	0.293 (0.188-0.455)	<0.05
GSTT1	Present	48 (20.9%)	176 (76.5%)	38.63	<0.001			1	
	Absent	182 (79.1%)	54 (23.5%)			12.36 (7.9-19.2)	<0.001	1.095 (0.629-1.903)	0.749

## Discussion

Lung cancer is a multifactorial disease influenced by both genetic and environmental factors, with genetic predisposition playing a crucial role in determining individual susceptibility. Among the various genetic contributors, polymorphisms in detoxification genes such as glutathione S-transferases (GSTs) - specifically, *GSTM1*, *GSTT1*, and *GSTP1 *- have been widely studied due to their involvement in the detoxification of carcinogens found in tobacco smoke and environmental pollutants. The GST enzyme family catalyzes the conjugation of glutathione to a wide range of electrophilic compounds, facilitating their elimination from the body. Therefore, alterations in these genes may lead to reduced detoxification efficiency and increased accumulation of DNA-damaging agents, which may contribute to carcinogenesis. Our findings demonstrate that the *GSTT1 *null genotype, *GSTM1 *null genotype, and *GSTP1 *variants (Ile105Val and Ala114Val) are significantly associated with an increased risk of lung cancer in the Maharashtra population we studied. The observed association suggests that individuals carrying these polymorphisms may have a reduced ability to neutralize carcinogenic metabolites, thereby predisposing them to higher genomic instability and cancer susceptibility. These results align with several studies of North American Caucasians, African-Americans, Mexican-Americans, Chinese, and Koreans conducted across diverse populations, which have consistently reported a link between GST polymorphisms and lung cancer risk [21-23].

The role of gene-environment interactions is also evident in our findings. The increased risk associated with these polymorphisms appears to be more pronounced among individuals with a history of smoking, emphasizing the synergistic effect between genetic susceptibility and exposure to tobacco-related carcinogens. Tobacco smoke contains a complex mixture of carcinogens, including polycyclic aromatic hydrocarbons (PAHs) and nitrosamines, which are substrates for GST-mediated detoxification. Variations in GST genes may, therefore, influence how efficiently these carcinogens are metabolized and excreted, ultimately affecting lung cancer risk [24].

The *GSTM1 *and *GSTT1 *genes, members of the multigene GST family, encode enzymes responsible for the detoxification of electrophilic compounds. Homozygous deletions in these genes (null genotypes) lead to complete loss of enzyme activity, impairing the body's capacity to detoxify carcinogenic compounds. According to Shukla et al., individuals with homozygous deletions in *GSTM1 *and *GSTT1 *are more susceptible to developing cancer because of their reduced ability to metabolize and eliminate carcinogens effectively [25].

Significant ethnic disparities have been observed in the prevalence of *GSTM1 *and *GSTT1 *null genotypes. The *GSTM1 *deletion occurs in nearly half of Caucasians and Japanese populations but is less common among African Americans, whereas the *GSTT1 *deletion is particularly prevalent in East Asian populations compared to Caucasian and African American groups [18, 26, 27]. These population-specific variations may contribute to differences in cancer susceptibility and underscore the importance of considering ethnic background in genetic association studies.

The *GSTP1 *gene encodes the pi-class glutathione S-transferase enzyme, which plays a critical role in detoxifying carcinogens, particularly those derived from tobacco smoke, such as benzo(a)pyrene and other PAHs. The *GSTP1 *exon 5 (Ile105Val) polymorphism results in an amino acid substitution that alters the enzyme’s substrate affinity and catalytic activity. The 105Val variant is associated with reduced enzyme efficiency, leading to decreased detoxification capacity and increased intracellular accumulation of carcinogenic metabolites. This reduced enzymatic activity has been linked to higher levels of DNA adduct formation and oxidative stress in lung tissue, which can contribute to tumour initiation and progression. Similar to the present study, Ramzy et al. found a significant association between the *GSTP1 *Ile105Val polymorphism and lung cancer susceptibility. They reported that individuals carrying the 105Val allele exhibited a higher risk of lung cancer than those with the wild-type 105Ile allele, supporting the potential role of this polymorphism in lung carcinogenesis [17].

Furthermore, the *GSTP1 *exon 6 (Ala114Val) polymorphism, although less studied, may also contribute to altered enzyme conformation and stability, further reducing detoxification efficiency. The combined effect of multiple GST gene variants (*GSTM1 *null, *GSTT1 *null, and *GSTP1 *polymorphisms) may act additively or synergistically, compounding the overall risk of lung cancer.

Despite the significant associations observed in the present study, several limitations should be acknowledged. First, this was a hospital-based case-control study conducted in a single region of southwestern Maharashtra, which may limit the generalizability of the findings to other populations with different genetic backgrounds and environmental exposures. Second, larger multicenter studies are needed to validate the results and improve statistical power. Third, only selected GST polymorphisms (*GSTM1*, *GSTT1*, *GSTP1 *exon 5, and *GSTP1 *exon 6) were evaluated, whereas other genetic variants involved in carcinogen metabolism and oxidative stress response may also contribute to lung cancer susceptibility. Fourth, environmental and occupational exposures were not assessed in detail, and smoking-related variables were not quantified in terms of duration or cumulative exposure, which may have limited a comprehensive evaluation of gene-environment interactions. Finally, the case-control design precludes the establishment of a causal relationship between GST polymorphisms and lung cancer risk. Therefore, prospective studies incorporating larger sample sizes, diverse populations, and comprehensive environmental exposure assessments are warranted to further clarify the role of GST polymorphisms in lung carcinogenesis.

## Conclusions

In summary, our study supports the hypothesis that genetic polymorphisms in detoxification enzymes play a significant role in modulating lung cancer risk, particularly in populations with high exposure to tobacco-related carcinogens. The presence of *GSTM1 *and *GSTT1 *null genotypes and *GSTP1 *variants suggests that these genetic variants may influence individual susceptibility to lung cancer. However, further large-scale, multi-centre studies incorporating comprehensive assessment of gene-environment interactions, lifestyle factors, ethnic diversity, and standardized quality-control procedures are required to validate these associations and clarify the underlying biological mechanisms. This comprehensive understanding of GST polymorphisms could aid in the development of population-specific cancer prevention strategies and contribute to personalized approaches in cancer risk assessment and management.

## Appendices

Lung cancer for patient information sheet

A] GENERAL INFORMATION:

1) Name of Patient:

2) Address:

3) Mobile Number:                                     

4) Religion:

5) Caste:                

6) Mother tongue: 

7) Details of family members:

B] PATIENT INFORMATION:

1) Date of birth:

2) Age and Gender of the patient:

3) Education:

4) Economic status:                                                                                                                                            

5) Family history of cancer: Yes/no

6) Family history of other diseases: Yes/no

7) History of alcohol drinking: Yes/no

8) Form of tobacco consumption:

9) Rate of tobacco consumption: Exclusive chewing/Exclusive smoking/Mixed habits/Habit free:

10) Duration, frequency, nature of tobacco habit:

11)  History of Smoking: Yes/no

12) History of Cigarette smoking: Never / Ever / Former / Current:

13) Dietary habits: Vegetarian/Non-vegetarian:

14) Intake of Vitamins or Minerals Supplements:

15) Use of oral contraceptives: Never/Ever/Missing data:

C] CLINICAL INFORMATION:

1. Clinical Symptoms:

2. Histopathology report:

3. Health Status:

4. Body indicators (Oral Symptoms):
